# Managing Illicit Online Pharmacies: Web Analytics and Predictive Models Study

**DOI:** 10.2196/17239

**Published:** 2020-08-25

**Authors:** Hui Zhao, Sowmyasri Muthupandi, Soundar Kumara

**Affiliations:** 1 Smeal College of Business Pennsylvania State University University Park, PA United States; 2 PricewaterhouseCoopers, LLP New York, NY United States; 3 Department of Industrial and Manufacturing Engineering Pennsylvania State University University Park, PA United States

**Keywords:** online pharmacy, web analytics, classification, illicit online pharmacies, online traffic analysis

## Abstract

**Background:**

Online pharmacies have grown significantly in recent years, from US $29.35 billion in 2014 to an expected US $128 billion in 2023 worldwide. Although legitimate online pharmacies (LOPs) provide a channel of convenience and potentially lower costs for patients, illicit online pharmacies (IOPs) open the doors to unfettered access to prescription drugs, controlled substances (eg, opioids), and potentially counterfeits, posing a dramatic risk to the drug supply chain and the health of the patient. Unfortunately, we know little about IOPs, and even identifying and monitoring IOPs is challenging because of the large number of online pharmacies (at least 30,000-35,000) and the dynamic nature of the online channel (online pharmacies open and shut down easily).

**Objective:**

This study aims to increase our understanding of IOPs through web data traffic analysis and propose a novel framework using referral links to predict and identify IOPs, the first step in fighting IOPs.

**Methods:**

We first collected web traffic and engagement data to study and compare how consumers access and engage with LOPs and IOPs. We then proposed a simple but novel framework for predicting the status of online pharmacies (legitimate or illicit) through the referral links between websites. Under this framework, we developed 2 prediction models, the reference rating prediction method (RRPM) and the reference-based K-nearest neighbor.

**Results:**

We found that direct (typing URL), search, and referral are the 3 major traffic sources, representing more than 95% traffic to both LOPs and IOPs. It is alarming to see that direct represents the second-highest traffic source (34.32%) to IOPs. When tested on a data set with 763 online pharmacies, both RRPM and R2NN performed well, achieving an accuracy above 95% in their predictions of the status for the online pharmacies. R2NN outperformed RRPM in full performance metrics (accuracy, kappa, specificity, and sensitivity). On implementing the 2 models on Google search results for popular drugs (Xanax [alprazolam], OxyContin, and opioids), they produced an error rate of only 7.96% (R2NN) and 6.20% (RRPM).

**Conclusions:**

Our prediction models use what we know (referral links) to tackle the many unknown aspects of IOPs. They have many potential applications for patients, search engines, social media, payment companies, policy makers or government agencies, and drug manufacturers to help fight IOPs. With scarce work in this area, we hope to help address the current opioid crisis from this perspective and inspire future research in the critical area of drug safety.

## Introduction

Online pharmacies (OPs) have grown tremendously in recent years, from US $29.35 billion in 2014 to an expected US $128 billion in 2023 globally, at an annual growth rate of 17.7% [1]. Most consumers pursue OPs for lower prices [2,3], convenience, and access to otherwise unavailable drugs, for example, recalled or on shortage [4,5]. However, there is insufficient awareness of the prevalent illicit online pharmacies (IOPs), which are estimated to represent 67%-75% web-based drug merchants [6]. Although much work has been carried out to restrict prescription for the recent opioid crisis, many IOPs provide access without prescription. IOPs provide unfettered access to prescription drugs and even controlled substances, leading to great concerns about substandard drugs, counterfeits, and supply chain integrity [7,8].

Fighting IOPs is critical in protecting patient safety as well as integrity of the drug supply chain. However, this is very challenging. First, there is low awareness of how to differentiate the legitimacy of OPs among consumers [9], and we still have much to learn about IOPs [6]. IOPs may look very similar to LOPs, and, unlike other consumer products, most consumers have no expertise in differentiating potentially substandard drugs even upon receiving them. Second, even identifying and tracking IOPs, the first step in fighting IOPs, can be challenging because of the sheer scale and the dynamic nature of the problem. According to Legitscript [6], there are 30,000-35,000 online pharmacies, and about 20 new IOPs are created when many *die* on a daily basis. Even if IOPs can be closed down (more difficult than we think as many IOPs have their servers outside of the United States), they can easily pop up using different URLs (eg, 30,000-35,000 known OPs represent only 2000-3500 merchants [6]).

A few checking systems of OP status (legitimate or illicit) do exist but with limitations. Some of them are *not* recommended [10], including the Canadian International Pharmacy Association and Pharmacychecker, which have been criticized for not always classifying the OPs correctly. The 2 sources recommended by the Food and Drug Administration are the National Association Board of Pharmacies (NABP) and Legitscript. However, both sources require consumers to take the initiative to look up the status of the pharmacies. According to a survey of 500 consumers from the United States, conducted by the Alliance for Safe Online Pharmacies, 95% do not know about the certification programs [9], let alone where to check the status of the OPs. Furthermore, there is no exhaustive database because of the aforementioned scale and the dynamic nature of OPs.

This study aims to use web analytics to better understand IOPs and to predict, identify and monitor IOPs using known information. We do this in 2 steps. First, we conducted a traffic analysis based on web-collected data, which assesses the means through which LOPs and IOPs are accessed and how engaged the customers are with them. On the basis of the information from the first step, especially through the analysis of referrals data, in the second step, we proposed a novel framework to predict the status (legitimate or illicit) of OPs based on the referral websites to them. Under this framework, we developed 2 easy-to-understand prediction models, the referral-based K-nearest neighbor (RKNN) and the referral rating prediction method (RRPM), and tested them using a data set with 763 OPs. We then implemented the 2 methods on Google search results for 3 popular drugs: Xanax (alprazolam), OxyContin, and opioids. These methods have many potential applications for consumers when shopping on the web and for other stakeholders to help fight IOPs, as presented in detail in the *Applications and Conclusions* subsection of the *Discussion* section.

## Methods

### Data Sources

We obtained the ground truth list of LOPs and IOPs from the NABP (Legitscript was not available for the size of our sample). NABP provided a list of approximately 1000 IOPs and 50 LOPs. We filtered out many IOPs that stopped operations at the time of data collection. We then collected usage data (ie, traffic and engagement data) for the remaining OPs from Similarweb and obtained the structure data (ie, referrals and backlink data, detailed later) from SEMrush. As Similarweb does not have data for websites not in its database or whose traffic is too low to monitor, this led us to the final sample sizes for each of the databases in Table 1. The first 5 rows in the table are the usage data, and the last row is the structure data. We collected data from Similarweb through web scraping using R. For SEMrush, we tried to collect the data manually (no crawling allowed for SEMrush). When that was impossible, we purchased the function from SEMrush (it sells different levels of functions through various priced accounts).

**Table 1:** Data sets and sample size.

Data set names	Legitimate pharmacies, n	Illicit pharmacies, n	Total samples, n	Data collection period
Traffic sources data	30	127	157	Average over 4 months (October 2015-February 2016)
Engagement data	30	127	157	Average over 4 months (October 2015-February 2016)
Country data	30	139	169	Average over 4 months (October 2015-February 2016)
Social media data	24	41	65	Average over 4 months (October 2015-February 2016)
Search data	30	60	90	Average over 4 months (October 2015-February 2016)
Referral data	50	713	763	September 2016

In Table 1, traffic sources provide the percentage of the sources through which consumers access the OPs, that is, direct, search, referral, social media, display, and email (details later). Engagement data show the extent of users’ involvement with the website (eg, the number of pages viewed and time spent on the website). Country data provide the percentage of traffic to OPs from different countries. *Social media data* refer to the proportion of traffic from 26 social media websites, such as Facebook, YouTube, and Google Plus. Search data provide the percentages of traffic resulting from organic or paid searches for OPs. An organic search, also called natural search, provides results by the search engine based on its relevance to the user’s query. Paid search results are like advertisements, where the websites pay search engines to promote their web pages for particular keywords. Referral data provide the different referring websites to online pharmacies, their internet protocol addresses, and countries of origin.

### OP Status Prediction Model

**Figure 1:**
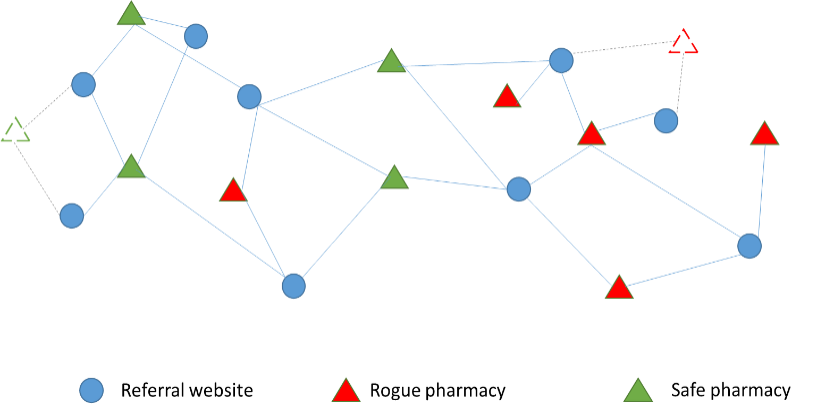
Simple Demonstration of Links Between Referral Websites and online pharmacies.

One of the difficulties in predicting the status of an OP is that the proposed method and the data it uses need to be something that cannot be easily manipulated by IOPs to affect future prediction results. To overcome this challenge, we propose a novel structure-based framework that predicts the status based on the relationship among the referral websites. Basically, we expect that if a pharmacy is mainly reached from referral websites that mostly link/refer to illicit pharmacies, then this pharmacy is more likely to be illicit. Figure 1 depicts an oversimplified demonstration of this idea and the links between referral websites.

To execute this idea, based on the ground truth list of LOPs and IOPs from NABP, we identified all the websites referring to the OPs in the data set and collected the structure data, that is, referrals and information of the number of backlinks to each OP, where a backlink is a link from a website to another website (eg, the OP here). These data, listed in Table 1 as the referral data, were then used to train the prediction model. Table 2 provides a snapshot of these data (the entries are the number of backlinks from a referral site j to a pharmacy i).

**Table 2:** Demonstration of our data set for the prediction model.

Pharmacy site, i	Referral site, j					
	1	2	3	...	j	...
1	5	0	3	…	16	…
2	9	3	0	…	0	…
i	0	0	0	…	2	…
...	…	…	…	…	…	…

**Figure 2:**
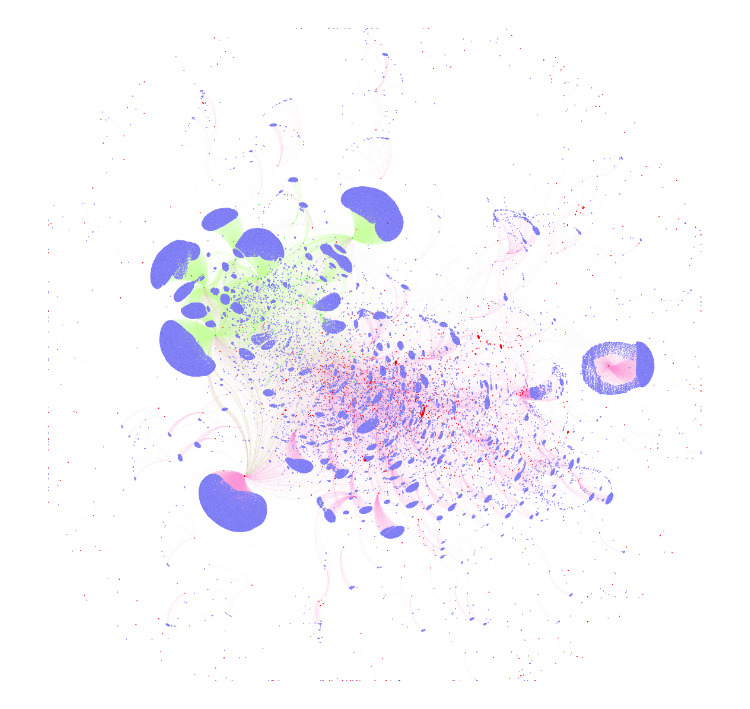
Relationship among referral websites and LOPs and IOPs based on real data.The pink nodes are the IOPs, the green nodes are the LOPs and the blue nodes are their referral websites. LOPs: legitimate online pharmacies; IOPs: illicit online pharmacies.

Figure 2 plots all our referral data. In the figure, the pink nodes are the IOPs, the green nodes are the LOPs, and the blue nodes are their referral websites. This figure shows 2 interesting phenomena: (1) LOPs and IOPs are clearly separated by the referral websites directing to them (although some referral websites refer to both IOPs and LOPs), that is, IOPs tend to be referred to by referring websites referring to other IOPs, and vice versa and (2) *good* referral websites tend to cluster in groups referring to each other’s referred pharmacies, whereas *bad* referral websites scatter around (they refer to all kinds of pharmacies far and between). These 2 phenomena, especially the first one, confirms our basic idea of using the *quality* of the referral websites (ie, how much the referring websites refer to LOPs) to predict the status of the OPs.

Then, we described in detail 2 prediction models that we developed based on this idea, that is, the RRPM and the RKNN.

#### RRPM

Let 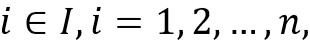 represent the set of pharmacies, 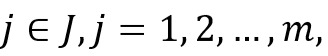 represent the set of referring websites, *l_ij_* represent the number of backlinks to pharmacy *i* from referring website *j*, and *y_i_* = 1 (*illicit*) or 0 (*licit*) represent the status of pharmacy *i.* We are now ready to present the model.

*Step 1: First define the quality of a referral website j (M_j_) based on its backlinks to legitimate and illicit pharmacies as*
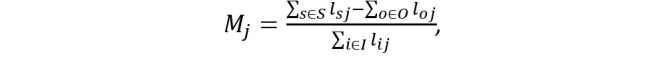
* where *
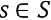
*represents the set of safe or legitimate pharmacies and *
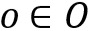
* represents the set of rogue or illicit pharmacies*.

Therefore, 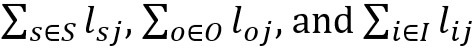 represent the number of LOPs, IOPs, and any OPs website *j* refers to, respectively. It is easy to see that *M_j_* is between –1 and 1 with *M_j_*=−1 indicating that website *j* only refers to IOPs and *M_j_*=1 indicating that website *j* only refers to LOPs.

*Step 2: For pharmacy i whose status is to be predicted, calculate the reliability score (R_i_)* as 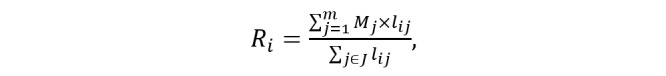 *where* 
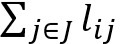
 *is the total number of backlinks (referral websites) to pharmacy i*. Note that it is possible that a given pharmacy to be predicted does not have any referral website from the training set. In this case, its *R_i_* will be indeterminate 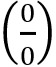 and *R_i_* is set to 0.

On the basis of our framework, we expect that the higher the *R_i_* is, the more likely it is legitimate. For our prediction model, we set a threshold T for the reliability score above which we predicted the pharmacy to be legitimate. In determining T, we considered a crucial factor, the sensitivity of the model, which measured the proportion of IOPs that were correctly identified as such. Although predicting a pharmacy wrong in either way is risky, for safety reasons, from the consumers’ perspective, classifying an illicit pharmacy as legitimate may be more detrimental than classifying a legitimate pharmacy as illicit. Taking this into consideration, we have the following:

*Step 3: Set the threshold T from the training set as *
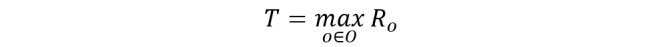
* such that we will classify pharmacy i as illicit if R_i_<T and legitimate otherwise*.

Notice that this is a very conservative threshold, meaning that a pharmacy is highly unlikely to be an illicit one when it is predicted legitimate. This could hurt the average accuracy of the model. Therefore, considering the average accuracy, one could select a different threshold. Another way we tried is to test different threshold levels with the training set and choose the one with the highest accuracy. Later, we reported the accuracy for both thresholds.

#### RKNN

In addition to RRPM, we next adapted one of the established classification methods, K-nearest neighbor (KNN), to our framework based on the referral links to develop another prediction model. KNN is a supervised learning model that classifies the samples in the test set based on their proximity to the samples of different classes in the training set [11,12]. The key to this method is defining proximity (similarity). We now incorporated our idea of the proposed framework into this definition.

*Step 1: Compute the Euclidean distance between the pharmacy x (the one whose status is to be predicted) and all the online pharmacies *i* with known status * i*=1,2,…,*n*, as* 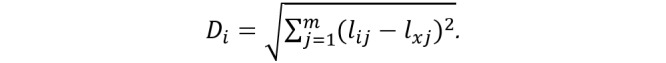 Note that the smaller the *D_i_* is, the more similar pharmacy x is to pharmacy *i* in terms of the referring websites directing to them.

*Step 2: Order the online pharmacies in decreasing order with respect to D_i_. Note down the status of the top K pharmacies. According to the traditional KNN, the status/class of x is assigned to the more frequent status among the K pharmacies. Formally, let the number of legitimate pharmacies among the top K be K_s_ and illicit ones be K_r._ We know that K_s_*+*K_r_=K. Let*
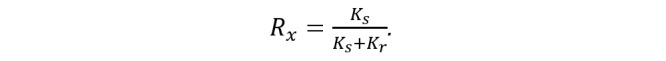
* x will be predicted to be legitimate if R_x_*>0.5. *R_x_* is similar to the reliability score of x, indicating the strength of the prediction, with a higher *R_x_* signifying a stronger prediction.

Similar to KNN, the performance of the RKNN model varies for different values of K. Obviously, a too high or too low value of K may reduce the accuracy of the model. We tested K=1, 2,…9 and reported the performance of the model for each value of K.

## Results

### Traffic and Use Analysis of the Online Pharmacies

Traffic sources of all websites are classified as direct, search, referral, social media, display, and email. Specifically, *traffic obtained by users’* directly typing in the URL of the website is classified as *direct*; *search* refers to the traffic coming from search engines such as Google, Bing, and Yahoo; traffic from links on other websites are accounted for as *referral*; *social* indicates the traffic from social media such as Facebook and Twitter; *display* indicates the traffic from banner advertising; and *Email* indicates the traffic coming from links in email messages.

Table 3 shows the mean percentage of traffic from each source to the IOPs and LOPs in our traffic data set (the standard deviation is shown in Multimedia Appendix 1). According to Table 3, direct, search, and referral are the 3 major traffic sources, representing more than 95% traffic to both LOPs and IOPs. A high percentage of direct traffic indicates that the specific OP website is a powerful brand and users visiting the website know what they want. Although LOPs are most accessed through direct traffic (42.48%), it is alarming to see that direct traffic also represents the second highest traffic source for IOPs (34.32%). This indicates that consumers who have previous experiences with IOPs (eg, from search, referrals) may become returning customers without knowing the aforementioned potential danger. Therefore, it is imperative to educate and alert patients, and curb people from using IOPs for the first time.

**Table 3:** Mean percentages of different traffic sources to online pharmacies.

Traffic source	Legitimate online pharmacy (n=30), %	Illicit online pharmacy (n=127), %
Direct	42.5	34.3
Search	36.3	39.3
Referral	17.7	21.7
Social	1.3	0.9
Email	2.2	0.6
Display	0	2.5

Previous research has indicated the presence of IOP contents on various social media sites [3]. Our data show that the average percentages of traffic through social media are less than 5% for both IOPs and LOPs, possibly because many OPs in our sample do not have traffic from social media. When we focused on the 24 LOPs and 41 IOPs from our sample that have substantial traffic from the 26 social media sites to conduct further analysis, we found that 92% (24/26) of the studied social media websites direct traffic to IOPs and only 50% (13/26) of them direct traffic to LOPs. Although 42% (11/26) of these social media websites direct traffic to both LOPs and IOPs, 50% (13/26) of the sites direct traffic to only IOPs and only 8% (2/26) of the websites direct traffic to only LOPs. Among the various social media (Table 4), we found that Facebook directs the highest traffic to both IOPs (58%) and LOPs (42%), far exceeding the second highest (Reddit), which directs 20% traffic to IOPs and 15% to LOPs.

**Table 4:** Traffic from social media websites to online pharmacies.

Social media	Proportion of traffic to legitimate pharmacies (n=24), %	Proportion of traffic to illicit pharmacies (n=42), %
Facebook	58	42
Reddit	15	20
YouTube	14	11
Twitter	4	—^a^
LinkedIn	2	—
Askville	—	7
Pinterest	—	4
Others	7	16

^a^Data negligibly small.

Furthermore, our country data (Table 5) show traffic from 52 countries for the 155 online pharmacies for which we were able to collect country data. Traffic from 27 (52%) countries points to only IOPs, and only 3 (6%) countries have traffic only to LOPs. In addition, the United States is the main consumer for online pharmacies, representing the highest proportion of traffic to both LOPs (97%) and IOPs (60%), among all countries.

**Table 5:** Traffic from different countries to online pharmacies.

Countries	Proportion of traffic to legitimate online pharmacies (n=30), %	Proportion of traffic to illicit online pharmacies (n=139), %
United States	97	71.1
Canada	1	—^a^
India	1	6.7
United Kingdom	—	7.6
Others	1	14.6

^a^Data negligibly small.

Table 6 shows the average engagement metrics across the LOPs and IOPs. This shows that the average monthly views (in millions), the number of pages viewed, and time spent on the sites of LOPs are all higher than those of IOPs, whereas the bounce rate (the percentage of visitors leaving the website after viewing only one page) is lower for LOPs than that for IOPs. This indicates that when consumers enter the OP websites, they seem to be more engaged with LOPs than IOPs. However, there are large variances in the monthly views of the websites, reflecting the huge differences among the websites in IOP as well as in LOP. For example, among the licit ones, cvs.com and walgreens.com are definitely the giants, whereas many others only attract a small number of views. In addition, although the average time spent on LOP sites (5 min) was significantly higher than that of IOPs (3.3 min) with *P*<.001, the maximum time spent on the sites of IOPs (17.4 min) was much higher than that on LOPs (10.6 min). This indicates that when consumers are interested, they may become very engaged with an IOP, leading to potential transactions. Hence, it is imperative to warn the patients before they enter a potential IOP, using the prediction method proposed by us.

**Table 6:** Consumers’ engagement with online pharmacies.

Types of the online pharmacies	monthly views in millions, mean (SD)	Number of page views, mean (SD)	Bounce rate, mean (SD)	Time on site in minutes, mean (SD)
Legitimate online pharmacy	1.48 (3.05)	7.2 (3.5)	32.2 (16.1)	5.0 (2.7)
Illicit online pharmacy	0.02 (0.05)	4.0 (2.1)	49.4 (17.9)	3.3 (2.2)

### OP Status Prediction Models

We now report the performance of the RRPM and RKNN models in their prediction. We consider 4 performance measures: accuracy, kappa, sensitivity, and specificity. Although sensitivity describes the percentage of IOPs correctly identified, specificity describes the percentage of LOPs correctly identified. We can see that *Type I error=1−specificity* and *Type II error=1−*sensitivity. As discussed, we chose the threshold T to pursue a minimum type II error, that is, a maximum sensitivity. In addition, the developed model should have good accuracy and reasonable kappa values, where kappa measures the agreement between observed and predicted classes considering to some extent the possibility of agreement by chance [13].

With 10-fold cross-validation [14], the performance metrics of RKNN (with K=1-9) and RRPM are shown in Table 7. It can be observed that all the RKNN models achieved 100% sensitivity. However, the specificity, accuracy, and kappa first increase and then decrease as K increases with R2NN performing the best, showing excellent metrics. RRPM also performs reasonably well, achieving a sensitivity of 99.2%, with relatively lower values for kappa and specificity. When changing the threshold T for RRPM from the current relatively conservative value to be the reliability score maximizing the model accuracy in the training data set, model accuracy, kappa, and specificity all we improve much. But sensitivity slightly dropped, as expected (Table 7).

**Table 7:** Performance of the classification models.

Model	Accuracy	Kappa	Specificity	Sensitivity
R1NN^a^	0.984	0.844	0.76	1
R2NN^a^	*0.986* ^b^	*0.859*	*0.78*	*1*
R3NN^a^	0.979	0.789	0.68	1
R4NN^a^	0.975	0.729	0.62	1
R5NN^a^	0.975	0.729	0.62	1
R6NN^a^	0.972	0.711	0.58	1
R7NN^a^	0.965	0.600	0.46	1
R8NN^a^	0.954	0.431	0.30	1
R9NN^a^	0.949	0.321	0.22	1
RRPM^c^	0.950	0.434	0.36	0.992
RRPM (alternative threshold)	0.968	0.648	0.78	0.977

^a^RKNN: reference-based K-nearest neighbor, where K=1-9.

^b^Indicates the best performing model.

^c^RRPM: reference rating prediction method.

### Implementing RRPM and RKNN on Google Search Results

Our traffic analysis showed that search accounts for the highest traffic to IOPs (39.27%). Our prediction model can be used in a couple of ways for search engines: (1) it can be incorporated on top of search results to filter/flag search results that are likely IOPs and (2) the reliability scores of the OPs can be used to rank the results such that more reliable OPs would appear first. Therefore, we tested our model on Google search results for 3 popular drugs.

Xanax (alprazolam) is a type of benzodiazepine. More than 30 percent of overdoses involving opioids also involve benzodiazepines [15]. Anecdotal evidence indicates that such drugs are typically the target of IOPs. We monitored the top keywords that direct traffic to OPs and identified that keywords with the drugs’ names contributed to more traffic than keywords without drug names. Hence, we chose *buy Xanax online* as the keyword and collected the top 100 search results for the keyword search on September 9, 2016. Almost all the search results were pharmacies selling Xanax on the web. As a result of the opioid crisis, along with *buy Xanax online*, we also studied the search results of the keywords *buy opioids online* and *buy OxyContin online* on April 22, 2017. OxyContin carries a boxed warning and contains oxycodone, a Schedule II controlled substance with an abuse potential similar to other Schedule II opioids.

To test our results, we hand collected the status of the OPs obtained through the top 100 search results from the NABP and Legitscript. Table 8 provides the status from both sources. Results demonstrate that neither source has an exhaustive database, although Legitscript (which only allows checking 10 pharmacies daily without a fee) has a bigger database, confirming what was found by Mackey et al [16]—that hand or automated search of opioid-related sites results in websites not covered by the Legitscript database. It is alarming to note that none of the pharmacies from the top 100 search results are legitimate by definition of either NABP or Legitscript. We then used RRPM and RKNN to predict the status of these pharmacies and compared our prediction results with the OP status according to Legitscript and NABP (Table 8).

**Table 8:** Status of the search results according to Legitscript and National Association Board of Pharmacies.

Keywords searched	IOP^a^ by NABP^b^	LOP^c^ by NABP	Unknown from NABP	IOP/rogue by Legitscript	LOP/safe by Legitscript	Unknown from Legitscript
Buy Xanax online	11	0	89	48	0	52
Buy Opioids online	6	0	94	34	0	66
Buy OxyContin online	10	0	90	25	0	75

^a^IOP: illicit online pharmacy.

^b^NABP: National Association Board of Pharmacies.

^c^LOP: legitimate online pharmacy.

As our model relies on the referral data, when the referral data for a particular online pharmacy is not available, its status is defined as unknown by our model. Table 9 compares the prediction results from RRPM and R2NN, respectively, with those from the Legitscript and NABP databases (NABP numbers are shown in parentheses) for the pharmacies obtained from the top 100 search results for the 3 keyword searches (hence, 300 overall). For instance, according to Table 9, 104 (27) pharmacies are correctly predicted illicit and 2 (0) are incorrectly predicted as legitimate pharmacies by RRPM when compared with the status defined by Legitscript (NABP). In addition, the status of 7 (0) IOPs according to the Legitscript (NABP) database cannot be identified by RRPM because of the lack of referral data.

**Table 9:** Comparison of the predicted status of online pharmacies based on reference rating prediction method (RRPM) and reference-based K-nearest neighbor (RKNN) with those obtained from Legitscript and National Association Board of Pharmacies (NABP) databases, with NABP numbers in parentheses.

Prediction results		Status obtained from Legitscript and NABP databases (NABP numbers in parentheses)		
		Illicit	Legitimate	Unknown
**Status estimated by RRPM^a^**				
	Illicit	104 (27)	0 (0)	147 (224)
	Legitimate	2 (0)	0 (0)	3 (5)
	Unknown	7 (0)	0 (0)	37 (44)
**Status estimated by R2NN^b^**				
	Illicit	106 (27)	0 (0)	145 (225)
	Legitimate	0 (0)	0 (0)	5 (5)
	Unknown	7 (0)	0 (0)	37 (43)

^a^RRPM: reference rating prediction method.

^b^R2NN: reference-based K-nearest neighbor.

Excluding those that are unknown from the corresponding databases, the tables show that RRPM and R2NN produced an error rate of 7.96% (0%) and 6.20% (0%), respectively, based on the Legitscript (NABP) database. The above results provide evidence that the proposed prediction models can predict online pharmacies with reasonably good accuracy.

## Discussion

### Comparison With Previous Work

In this study, we conducted a web traffic and engagement analysis of IOPs and LOPs, developed simple prediction models of the status of the OPs based on referral links, and tested the prediction models with data for 763 online pharmacies. Although the previous literature shows evidence of drug selling through IOPs, there has been very limited work on the traffic to these websites. One exception is the study by Mackey et al [5], which estimated traffic to an IOP through fictitious advertisements for selling drugs without prescription that they created on social media. In contrast, we collected true data on the traffic analysis and the prediction models.

Similarly, very limited research is related to identifying and predicting the status of OPs. The study by Fittler et al [10] aimed to identify the indicators of IOPs by evaluating 136 of them based on the longevity of the site, geographical location, display of contact information, medical information exchange, prescription requirement, and pharmacy legitimacy verification. They identified that the prescription requirement or availability of contact information does not correlate with illicit pharmacy status as indicated by Legitscript; however, the long-term continuous operation of the website has a strong correlation with illicit activities. They did not develop a prediction model.

Predicting the status of OPs is related to classifying different websites into certain categories. In general, there are 2 types of approaches: content based and structure based. Hybrid methods also exist. While content-based classification [17] utilizes the website content to classify the website, structure-based classification exploits the patterns in the link structure or the topology of the hyperlinks of the websites. For example, Amitay et al [18] used structural information to classify 8 classes of websites (eg, corporate sites, search engines, and e-store), with the precision of certain classes exceeding 85%. Our prediction model is structure based, and it is easy to see what we try to classify as IOP and LOP is much more subtle.

Research on the prediction/classification of LOPs and IOPs is very scarce. The only other work is the study by Corona et al [19] aimed at building a database of OPs using textual content analysis. Note that content-based prediction could be more easily manipulated than structure-based prediction. For example, if the prediction is based on certain content appearing on the websites, then IOPs could delete or change the content to confuse the model by making it just *like* LOPs. Toward this end, this paper proposes a novel yet simple structure-based idea using relationships among referral websites to predict the status of OPs.

Finally, when searching the literature for general prediction and classification of websites selling counterfeit products (not limited to drugs), only 2 studies were found [20,21]. Both used content analysis in general, achieving an accuracy of 86.4% [20] and 88% [21]. Our approach can potentially be applied to more general products than just drugs.

### Limitations

As we propose a new methodology, we face many limitations. First, because of the limited source of the available ground truth of the status of online pharmacies and the data related to traffic analysis, we have a relatively small sample size (for some of the traffic analysis). We expect that a larger sample size when available will improve the accuracy of the results and allow more detailed analysis. When using Google search results, we also face many websites whose true status is unknown; hence, evaluation of our methods using the Google search results presented in our paper is limited. Second, the current website information sources (SEMRush and Similarweb) do not provide reliable (or any) information for small websites lacking sufficient data for traffic overview. Accordingly, the findings of this research are mostly applicable to larger legitimate and illicit web-based players. Third, our proposed method relies on referral website data. Our current referral database from the data we collected seemingly works well. However, obviously, the bigger the referral link database, the better. Although outdated links do not hurt the performance (they will not be used), updating these links as more ground truth data becomes available would be desired. Finally, we focus on proposing a novel structure-based prediction framework and developing simple models to help resolve an important and practical problem. More advanced models, such as a hybrid of structure based and contexture based, can be developed in the future to further improve performance.

### Applications and Conclusions

Previous research shows that illicit online pharmacies are present and widely accessed, posting dramatic risks to the drug supply chain integrity and patient health. However, because of the sheer scale of this problem (>30,000 OPs) and the dynamic nature of online channels, even identifying and monitoring IOPs, the first step to curb IOPs, is a difficult task. In this study, we aimed to fill this gap by conducting a traffic analysis to increase our understanding of IOPs and proposed a new idea to predict the OP status based on referral data and developed 2 specific prediction models (RRPM and RKNN) using this idea. Testing these models on a data set with 763 online pharmacies showed that both models performed well, with an accuracy of 95.0% (RRPM) and 98.6% (RKNN). R2NN outperformed RRPM in more comprehensive metrics (sensitivity, kappa, and specificity). When implementing both models on the Google search results for 3 drugs, we only incurred an error rate of 6.20% for the pharmacies whose true status was known according to the Legitscript database when using the R2NN model and an error rate of 7.96% when using RRPM for the prediction. Although further testing with a larger data set is being pursued (the difficulty is the limited ground truth data), we believe our traffic analysis and the approach to use web analytics of referral websites to predict the status of OPs is among the first in the drug field and proposes a viable and evidently effective way to monitor OPs.

The developed framework/models have numerous exciting application areas. For example, they can be implemented by search engines, social media, web-based markets (eg, Amazon), and payment companies (eg, Visa and Master cards) to filter IOPs or take the status of the online pharmacies into consideration when ranking search results, deciding advertising allocations, making payments, disqualifying vendors, or at least warning consumers of potential IOPs. They can also be used with search engines and social media to develop a warning system to help consumers make informed decisions. The timeliness of this work could help address the current opioid crisis. Policy makers, government agencies, patient advocacy groups, and drug manufacturers may also use such a system to identify, monitor, curb IOPs, and educate consumers.

Given that this is a critical area of concern to patients’ health and the integrity of the drug supply chain, we hope this study will inspire additional efficient and effective prediction models or additional applications for the prediction models developed. On a larger scale, we hope to inspire more research in other aspects to fight IOPs. Finally, our literature review also reveals that literature on automatic prediction/identification of websites selling counterfeit products (not limited to drugs) is also very scarce, although selling counterfeit products on the web is a prevalent problem. Our framework and prediction models can be applied to other products, and we hope to inspire research in this general area as well.

## Appendix

Multimedia Appendix 1 More details of the engagement and traffic source data.

## Abbreviations

IOP: illicit online pharmacies
KNN: K-nearest neighbor
LOP: legitimate online pharmacies
NABP: National Association Board of Pharmacies
RKNN: reference-based K-nearest neighbor
RRPM: reference rating prediction method
