# Supplementary material for: Managing Illicit Online Pharmacies: Web Analytics and Predictive Models Study
Source: J Med Internet Res. 2020 Aug 25;22(8):e17239. doi: 10.2196/17239 (PMC7479587; doi:10.2196/17239)
Supplement: Multimedia Appendix 1 [file jmir_v22i8e17239_app1.docx]

Appendix More Details of the Engagement And Traffic Source Data

| IOP | Views  (millions) | | page views | | | bounce rate | | Time on site (mins) | | Direct | referral | | | search | | | | | | social | | email | | display | |  |  |  |
| --- | --- | --- | --- | --- | --- | --- | --- | --- | --- | --- | --- | --- | --- | --- | --- | --- | --- | --- | --- | --- | --- | --- | --- | --- | --- | --- | --- | --- |
|  |  | |  | | |  | |  | |  |  | | |  | | | | | |  | |  | |  | |  |  |  |
| mean | 0.02 | | 4.0 | | | 49% | | 3.3 | | 34% | 22% | | | 39% | | | | | | 1% | | 1% | | 2% | |  |  |  |
| stdev | 0.05 | | 2.1 | | | 18% | | 2.2 | | 22% | 21% | | | 29% | | | | | | 2% | | 1% | | 12% | |  |  |  |
|  |  | | |  | | |  | |  | | | |  | | |  |  |  | | | | | | | | |  |  |
|  |  | | |  | | |  | |  | | | |  | | |  |  |  | | | | | | | | |  |  |
|  |  | | |  | | |  | |  | | | |  | | |  |  |  | | | | | | | | |  |  |
| LOP | Views (millions) | page views | | | bounce rate | | | | Time on site (mins) | Direct | | referral | | | search | | | | social | | email | | display | |  |  |  |  |
| mean | 1.48 | 7.2 | | | 32% | | | | 5.0 | 42% | | 18% | | | 36% | | | | 1% | | 2% | | 0% | |  |  |  |  |
| stdev | 3.05 | 3.5 | | | 16% | | | | 2.7 | 16% | | 9% | | | 20% | | | | 4% | | 3% | | 0% | |  |  |  |  |
